# Alternative splicing dynamics in pig tissues under soybean oil supplementation

**DOI:** 10.1007/s00335-026-10279-4

**Published:** 2026-10-15

**Authors:** Fernanda Nery Ciconello, Bruna Pereira Martins da Silva, Milena Lucia Cornejo, Vivian Vezzoni de Almeida, James Eugene Koltes, Aline Silva Mello Cesar

**Affiliations:** 1 Department of Animal Science, Luiz de Queiroz College of Agriculture, Piracicaba, Brazil; 2 Department of Animal Science, School of Animal Science and Food Engineering, Pirassununga, Brazil; 3 School of Veterinary Medicine and Animal Science, Goiás Federal University, Goiânia, Brazil; 4 Department of Animal Science, Iowa State University, Ames, IA USA; 5 Department of Food Science and Technology, Luiz de Queiroz College of Agriculture, Piracicaba, Brazil

## Abstract

Lipid metabolism in pigs is a relevant model for understanding human metabolic regulation due to physiological similarities between the species. Alternative splicing (AS) generates transcript variants that can modulate metabolic pathways in the brain, liver, and skeletal muscle. This study aimed to identify and characterize AS events to explore the dynamics of AS under supplementation of soybean oil in these three tissues. We performed RNA sequencing in 36 pigs fed a soybean–corn diet supplemented with 1.5% or 3.0% soybean oil, using brain, liver, and muscle tissues. Expression matrices and structural annotation were generated for SUPPA analyses. Seven classes of splicing events were identified for each tissue, and only candidate genes exhibiting the highest differential proportion of spliced-in (DPSI) values were considered. Events were detected in both coding regions and 5’ UTRs, indicating potential functional consequences. Differential splicing was present in *FRMPD1* for brain, *G6PC3* for liver, and *MED1* and *FBXL20* for muscle. These findings suggest that AS may regulate lipid metabolism and that coordinated splicing responses across tissues could also influence the metabolic adaptation and growth performance.

**Supplementary Information:**

The online version contains supplementary material available at https://doi.org/10.1007/s00335-026-10279-4.

## Introduction

Modern pig production faces the challenge of balancing productivity, meat quality, and animal health, while also seeking to meet the nutritional and functional demands of the end consumer (Mote and Rothschild 2019). In this context, modulating lipid metabolism through diet has emerged as a central strategy, particularly through the supplementation of lipid sources, such as soybean oil, which is widely used in swine diets in Brazil and around the world (Fanalli et al. 2022b, 2023; Ciconello et al. 2026b).

Body fat composition in pigs, and consequently that of pork, is strongly influenced by diet, making it possible to modify the fatty acid profile through the supplementation of different sources and levels of vegetable oils (Vehovský et al. 2015; Alencar et al. 2021). Soybean oil, which is rich in polyunsaturated fatty acids, especially linoleic acid (C18:2, n-6), has the potential to increase the concentration of this fatty acid in pork fat, thus impacting not only the nutritional quality of the meat but also the metabolic and physiological aspects of pigs (Vehovský et al. 2019). Furthermore, modulation of the lipid profile can influence inflammatory and immunological processes as well as energy metabolism, with direct effects on growth performance and the overall health of pigs (Ciconello et al. 2025, 2026b).

The relevance of the pig as an experimental model for studies of lipid metabolism and human metabolic diseases arises from its anatomical, physiological, and metabolic similarity to humans (Lunney et al. 2021). In this regard, understanding how different levels of soybean oil modulate gene expression and the underlying molecular mechanisms, such as alternative splicing, is fundamental to advancing nutrigenomics applied to animal production and public health (Fanalli et al. 2022a; da Silva et al. 2023; Ciconello et al. 2026a).

Alternative splicing (AS) is one of the main post-transcriptional mechanisms of gene expression regulation in eukaryotes, allowing a single gene to encode multiple mRNA isoforms and, consequently, different protein variants (McManus and Graveley 2011; Thakur et al. 2018; Scoville et al. 2025). It is estimated that more than 90% of human multi-exonic genes undergo AS, a phenomenon that is also widely observed in pigs and other mammals (Feng et al. 2024). This mechanism contributes to proteomic diversity, cellular plasticity, and adaptation to diverse physiological and environmental conditions (Le et al. 2015; Scoville et al. 2025).

The sensitivity of AS to metabolic changes is particularly relevant in metabolically active tissues, such as the brain, liver, and skeletal muscle. The brain is one of the tissues with the greatest diversity of transcriptional isoforms, and AS plays a role in neurogenesis, synaptic plasticity, and response to environmental stimuli (Deng et al. 2023). The liver, a central organ in lipid metabolism, exhibits a wide range of AS events that regulate candidate genes involved in fatty acid oxidation, lipoprotein synthesis, and inflammatory responses (Jobbins et al. 2023). Skeletal muscle, in turn, has AS that modulate candidate genes associated with fatty acid uptake and oxidation, energy metabolism, and adaptation to exercise or nutritional stress (Gaugel et al. 2025).

The SUPPA tool, in particular, has established itself as a reference tool for large-scale AS analysis due to its ability to calculate percent spliced-in (PSI) values directly from transcript abundance quantified by rapid methods, including RSEM, Salmon, Kallisto, or Sailfish. Recent comparative studies show that SUPPA performs as well or is better than other tools in accuracy, robustness to technical variation, and scalability, and can process thousands of samples in minutes with low memory consumption (Trincado et al. 2018).

Despite advances in bioinformatics tools for analyzing alternative splicing, challenges remain in detecting novel events, accurately quantifying low-abundance isoforms, and functionally interpreting the results. Most tools rely on genomic annotations, which can limit the detection of unannotated isoforms, especially in non-model species or poorly studied tissues. Our understanding of splicing mechanisms and their regulation is advancing, but further studies are still needed to develop a deeper understanding of how splicing regulation functions in both protein-coding and non-coding candidate genes. Consequently, this work will help fill this gap in splicing research. The present study not only demonstrates the possibility of detecting unannotated isoforms but also establishes that annotated isoforms exhibit notable differential splicing, suggesting that these annotated isoforms may regulate important metabolic processes in three key porcine tissues. Thus, this study aimed to identify and characterize alternative splicing (AS) events in different pig tissues and explore how dietary soybean oil could be influenced by these events, potentially altering protein structure or regulating gene expression based on their location within affected and candidate genes.

## Materials and methods

### Animals and sample collection

A total of 36 purebred Large White immunocastrated male pigs (*Sus scrofa*), offspring of 32 sows and three sires, with an average initial body weight of 28.05 ± 3.17 kg and an average age of 71 ± 1.8 days, were used in a 98-day study. The pigs were randomly assigned to one of two dietary treatments: a soybean-corn meal base supplemented with either 1.5% soybean oil (SOY1.5) or 3.0% soybean oil (SOY3.0). The SOY1.5 is considered the control group because this diet is commercially used for pigs. All animals were genotyped for the halothane mutation (*RYR1* gene), and all were halothane homozygous negative (NN) (van Arendonk and Kennedy 1990). Each pen had a hole-dry self-feeder and a nipple waterer, allowing pigs *ad libitum* access to feed and water during the experiment (Almeida et al. 2021). The representative diet sample was obtained from each batch and then pooled by treatment in a composite sample for fatty acid composition analysis (Table 1), which was performed as described by Almeida et al. (2021).

**Table 1:** Analyzed fatty acids of finisher diets SOY1.5 and SOY3.0 (as-fed basis). Adapted from Almeida et al. (2021)

Fatty acid, %	Diet (day 42 to 98)	
	SOY1.5	SOY3.0
Saturated fatty acid (SFA)		
Myristic acid (C14:0)	0.32	1.08
Palmitic acid (C16:0)	13.25	14.62
Margaric acid (C17:0)	0.15	0.25
Stearic acid (C18:0)	4.07	3.92
Arachidic acid (C20:0)	0.44	0.43
Behenic acid (C22:0)	0.29	0.21
Monounsaturated fatty acid (MUFA)		
Palmitoleic acid (C16:1)	0.27	0.88
Oleic acid (C18:1, n-9)	32.02	32.83
Eicosenoic acid (C20:1, n-9)	0.30	0.24
Polyunsaturated fatty acid (PUFA)		
Linoleic acid (C18:2, n-6)	45.97	43.23
Alpha-linolenic acid (C18:3, n-3)	3.48	2.98
Eicosapentaenoic acid (C20:5, n-3)	ND^3^	ND
Docosahexaenoic acid (22:6, n-3)	ND	ND
Total SFA	18.52	20.51
Total MUFA	32.59	33.95
Total PUFA	49.45	46.21
PUFA: SFA ratio^1^	2.67	2.25
∑n-6/∑ n-3 PUFA ratio^2^	13.21	14.51

^1^PUFA: SFA ratio = total PUFA/ total SFA

^2^∑n-6/∑ n-3 PUFA ratio = linoleic acid / alpha-linolenic acid

^3^ND = non detectable

All diets were formulated to meet established nutrient requirements according to Rostagno et al. (2011), as described in Almeida et al. (2021). None of the diets contained antimicrobial growth promoters. According to the manufacturer’s recommendations, pigs were immunocastrated through 2 mL doses of Vivax^®^ (Pfizer Animal Health, Parkville, Australia) at 105 days and 133 days of age. After 98 days of the trial, pigs were slaughtered with a final age of 169 ± 7 days and an average final body weight of 131.68 ± 8.6 kg. After exsanguination, we collected samples of the brain, liver, and *Longissimus (L.) lumborum* muscle, snap-froze them in liquid nitrogen, and stored them at − 80 °C for subsequent RNA-sequencing (RNA-Seq) analyses. More details are provided in Almeida et al. (2021), Fanalli et al. (2022), and Silva et al. (2023). All studies followed ARRIVE guidelines 2.0 (du Sert et al. 2020).

### Data analysis

All the sequencing analyses were performed at the Genomic Center in the Animal Biotechnology Laboratory at ESALQ/USP, Piracicaba, São Paulo, Brazil. Total RNA was extracted from the brain, liver, and skeletal muscle using the RNeasy Mini Kit (QIAGEN, Hilden, Germany) following the manufacturer’s instructions. Quantification, purity, and integrity of total RNA were evaluated by the Nanodrop 1000 and Bioanalyzer 2100. The RNA integrity number (RIN) was between 7.0 and 10.0 for all samples. To prepare the library, 2 µg of total RNA was used according to the TruSeq RNA Sample Preparation Kit v2 Guide protocol (Illumina, San Diego, CA). The average library size was determined using an Agilent Bioanalyzer 2100 (Agilent, Santa Clara, CA, USA). The quantification of the libraries was performed using quantitative PCR with the KAPA Library Quantification Kit (KAPA Biosystems, Foster City, CA, USA). The quantified samples were diluted, labeled with a barcode, and pooled to be run in different lanes using the TruSeq DNA CD Index Plate (Illumina, San Diego, CA, USA). All the samples were sequenced across five lanes on the flow cell using the TruSeq PE Cluster Kit v4- cBot-HS kit (Illumina, San Diego, CA, USA). Then, samples were clustered and sequenced using HiSeq 2500 equipment (Illumina, San Diego, CA, USA) with a TruSeq SBS Kit v4-HS (200 cycles) according to the manufacturer’s instructions.

The quality of the raw RNA-Seq reads was checked by FastQC version 0.11.8 [http://www.bioinformatics.bbrc.ac.uk/projects/fastqc/]. Due to the presence of adaptors, indexes, low complexity reads, and barcodes, TrimGalore version 0.6.5 [http://www.bioinformatics.babraham.ac.uk/projects/trim _galore] was used to remove them. We added a filter higher than 33 for Phred score and another higher than 70 nucleotides for read length to the following steps. The alignment was made with the pig reference genome *Sus scrofa* 11.1, available at Ensembl [http://www.ensembl.org/Sus_scrofa/Info/Index]. The alignment, mapping, and abundance (read counts) of mRNAs were performed using STAR version 2.7.6a (Dobin and Gingeras 2015). The structural annotation was conducted using StringTie version 2.2.1 to generate a Gene Transfer Format (GTF) file for input into the differential splicing analysis. In StringTie, the GTF files were merged using the merge function, and the following parameters were used: a minimum transcript length of 50 bp, a TPM threshold of 0.1, and coverage equal to 97.68 for brain, 97.32 for liver, and 95.60 for skeletal muscle, similar to the RNA-Seq coverage obtained for each tissue.

The read counts for all annotated mRNAs were aligned using Bowtie2 version 2.4.3. We normalized gene expression levels using transcripts per million (TPM) calculated by RNA-Seq by Expectation–Maximization (RSEM) version 1.3.1 (Trincado et al. 2018a). This method estimates the number of fragments originating from each gene in each replicated library, effectively handling reads that map ambiguously between isoforms and candidate genes and reducing variation in total read counts across samples (Li and Dewey 2011).

### Differential splicing analysis

Differential splicing analysis is fast, precise, and conscientious of uncertainties on multiple conditions. For this analysis, Python version 3.12 was installed, and the SUPPA software (version 2.3) was installed (Alamancos et al. 2015; Trincado et al. 2018). SUPPA has three subcommands: generateEvents, psiPerEvent, and diffSplice. Each one is explained as follows: generateEvents can generate events following one annotation file; psiPerEvent quantifies the level of event inclusion in several samples; and diffSplice calculates the differential splicing in multiple conditions (SOY1.5 and SOY3.0) with replicates (36 animals).

The workflow is followed by the subcommand generateEvents. We used specific arguments, including a mandatory one to specify the output file format (e.g., ioe), as well as two arguments: event type and pool genes. Next, we used the psiPerEvent subcommand, which also included arguments such as total filter equal to 0.1. For the diffSplice subcommand, we used several arguments, including empirical method, area equal to 1,000, lower bound greater than 0.05, and gene correction, which corrects each gene’s *p*-value for FDR.

The AS events were generated from an annotation GTF file created with StringTie using the merge function. This method reads only the “exon” transcript information. It can generate events and produce a single file of events with the extension “ioe” for local AS events. Specifically, during PSI calculation, we used transcripts that contribute to the numerator (one alternative of the event) and the denominator (both alternatives of the event).

**Fig. 1:**
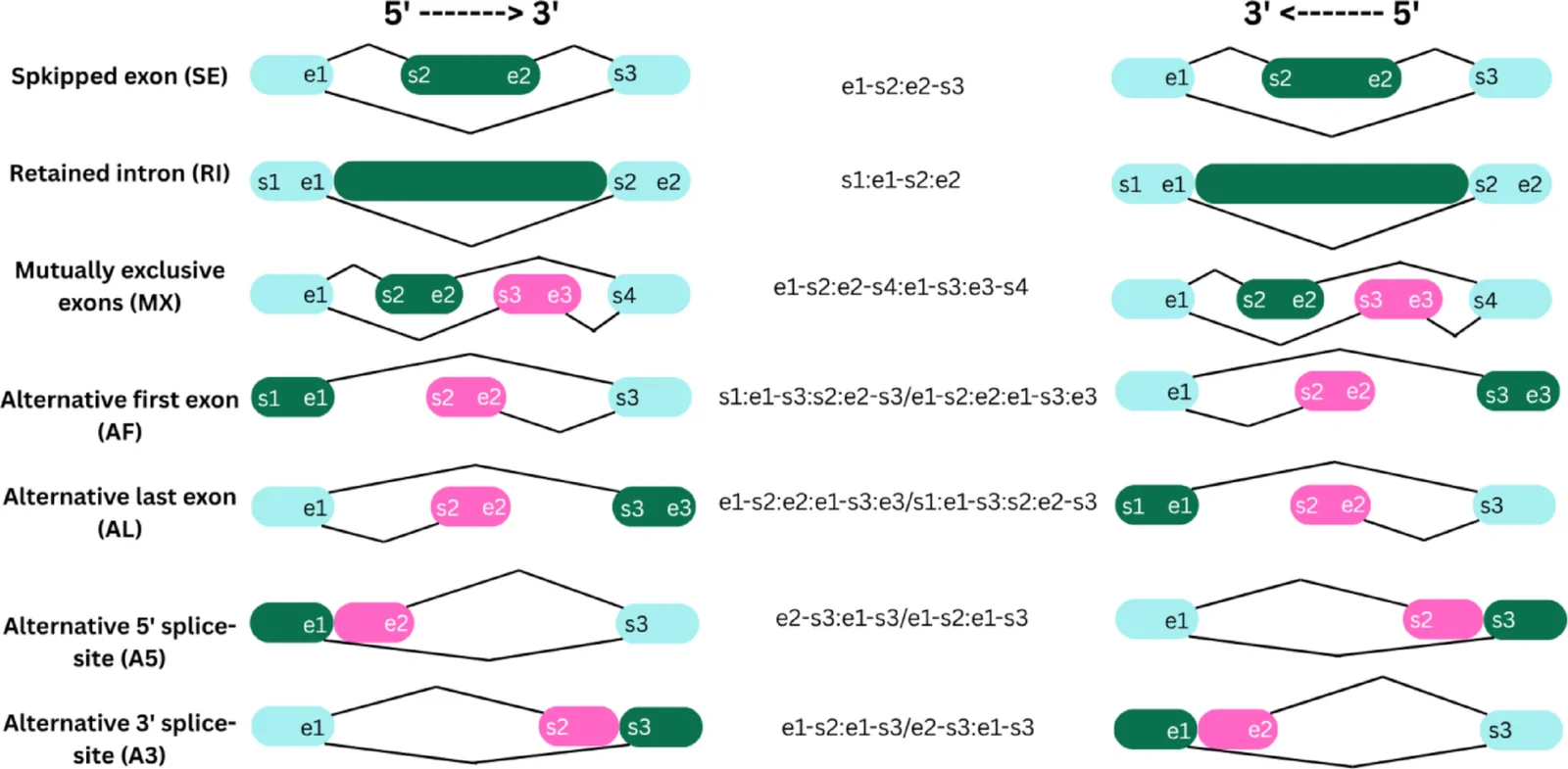
The figure describes the event names in the sense (5’→3’) and antisense (3’←5’) strands, respectively. Each event is identified by a single form, defined by a set of coordinates: the start (s) and end (e) coordinates for the different exon regions involved in the event. The external coordinates from the event are used only for RI, AF, and AL. The AS event form that includes one dark green region is used to calculate the relative inclusion level. The pink area notes the other alternative form of the event. For example, for RI, the inclusion level is assigned to the form that retains the intron

The different local events generated by SUPPA can be described in Fig. 1:

- Skipped Exon (SE).
- Alternative 5’/3’ Splice-Sites (A5/A3) (generated along the option SS).
- Mutually Exclusive Exons (MX).
- Retained Intron (RI).
- Alternative First/Last Exons (AF/AL) (generated along the option FL).

The argument pool-genes was used to create “ioe” file following the annotations non-based in loci. This argument is strongly recommended for Ensembl and Gencode, which contain genes with overlapping transcripts that share large parts of the sequence, so their relative contribution to AS events is unclear. The exon-length option was maintained as the default. The transcript expression output (calculated in TPM) was filtered to remove 80% of samples with no expression among samples in the same treatment; this filtering step was performed for each treatment group (e.g., SOY1.5 and SOY3.0).

For the PSI calculation for AS events, SUPPA reads the “ioe” file to calculate the relative abundance (relative percentage between transcript abundance in a specific sample and the abundance of N samples) per sample for each local event. The output file is given with a “PSI” extension for each expression file (SOY1.5 and SOY3.0). PSI values are normalized between 0 and 1.

To improve the robustness of the computational evidence, we applied stringent criteria for transcript abundance, DPSI, statistical significance, and biological replicates. In this way, we also performed differential splicing analysis for local events across multiple samples, keeping all default settings. We only added one argument: gene-correction, which indicates FDR correction by candidate gene. The events considered for evaluation should have an FDR lower than or equal to 10% due to the specificity and sensitivity declared by the developers of the SUPPA tool and according to other authors who restricted the data to analyze in a biological relevance the candidate genes which presented differential splicing (Alamancos et al. 2015b; Solomon et al. 2017; Trincado et al. 2018b; Mertes et al. 2021; Wen et al. 2023; Shorrock et al. 2024). These events had an FDR of < 10%, and we annotated the highest DPSI using Ensembl BioMart (version 115) with the *Sus scrofa* 11.1 genome (Dyer et al. 2025). To verify the influence of the AS event on translation, we collected sequences from UniProt (Bateman et al. 2021) and used the NCBI BLASTp online tool (Altschul et al. 1990). The GTF file generated by the script generateEvents can be viewed in the IGV browser(Thorvaldsdóttir et al. 2013) to identify the region where the AS event is occurring and to search Ensembl to determine which exon the AS event is inserted into (Dyer et al. 2025). After analyzing the similarity indices, we integrated BLASTp with Ensembl Viewer to determine if the AS is happening in the coding sequence (CDS) or untranslated region (UTR) of a significant gene (FDR ≤ 0.1) and high |DPSI|. If AS occurs in a CDS region, the protein 3D structure was generated using ChimeraX (v. 1.10.1) (Pettersen et al. 2021).

### Functional enrichment

The functional enrichment analysis was carried out on the web-based Gene Set Analysis Toolkit (WebGestalt) using over-representation analysis for the organism of interest, *Sus scrofa*, and the functional database Gene Ontology (GO), including only biological processes, with no redundancy. All the candidate genes that possessed an AS event could be enriched in each tissue and can be viewed in the results section. The gene list input included annotated genes, independent of the AS event (Supplementary Table S1).

## Results

The differential splicing analysis was based on two conditions, SOY1.5 and SOY3.0, used to analyze the AS event in three pig tissues. The DPSI varies from − 1 to 1. Positive values of DPSI indicate the probability of AS occurring in SOY1.5, or negative values indicate the probability of AS occurring in SOY3.0 for annotated genes. Non-annotated genes have received a code from StringTie. However, we could not perform functional annotation on Ensembl, functional enrichment on WEBGestalt, or protein design on ChimeraX for these non-annotated genes.

### Alternative splicing events in three tissues

**Fig. 2:**
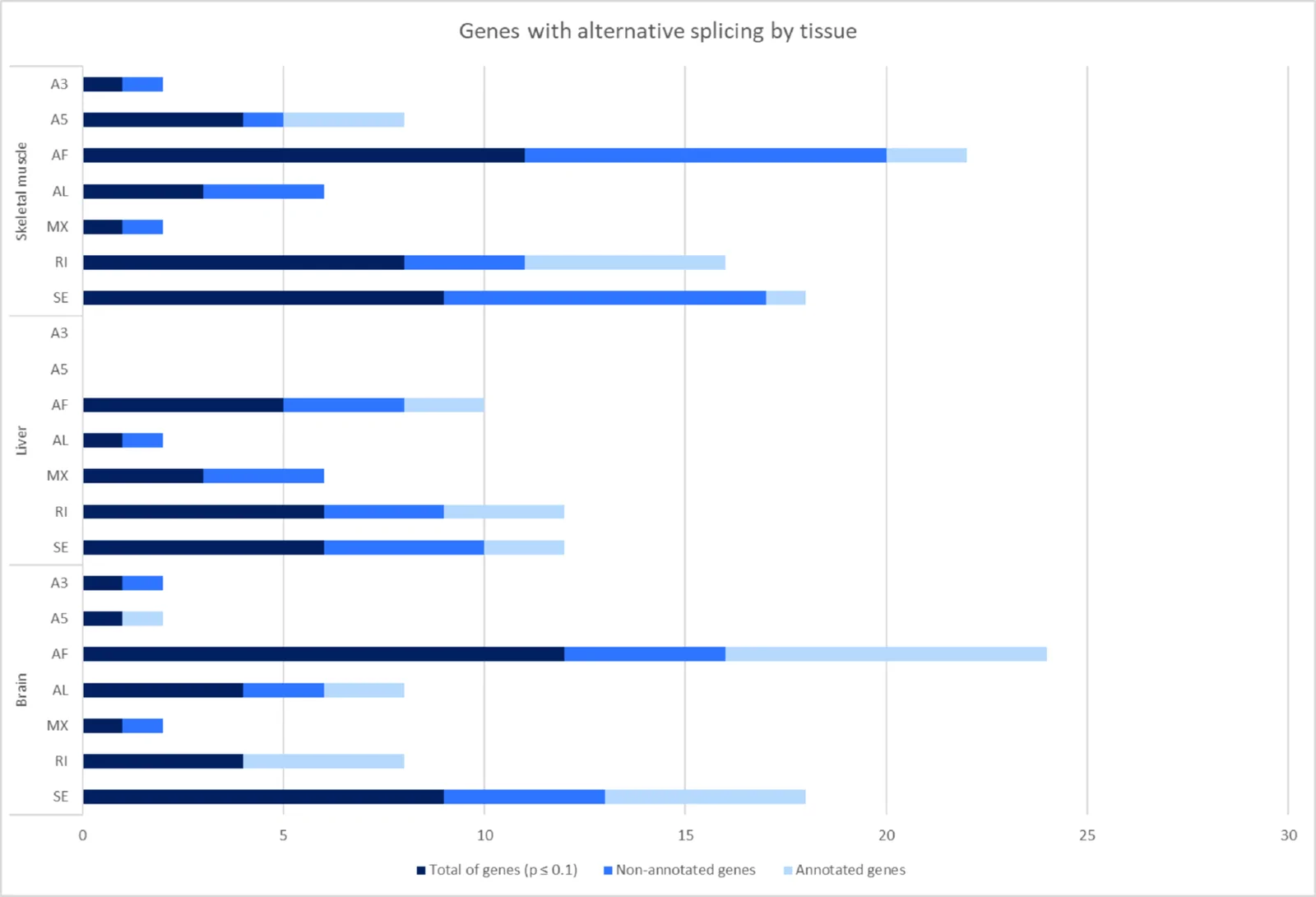
Total candidate genes with differential splicing (FDR ≤ 0.1) for the three tissues analyzed under the two diet conditions (SOY1.5 and SOY3.0). The events are *SE* skipped exon, *RI* retained intron, *MX* mutually exclusive exons, *AF* alternative first exon, *AL* alternative last exon, *A5* alternative 5’ splice-site and *A3* alternative 3’ splice-site

**Fig. 3:**
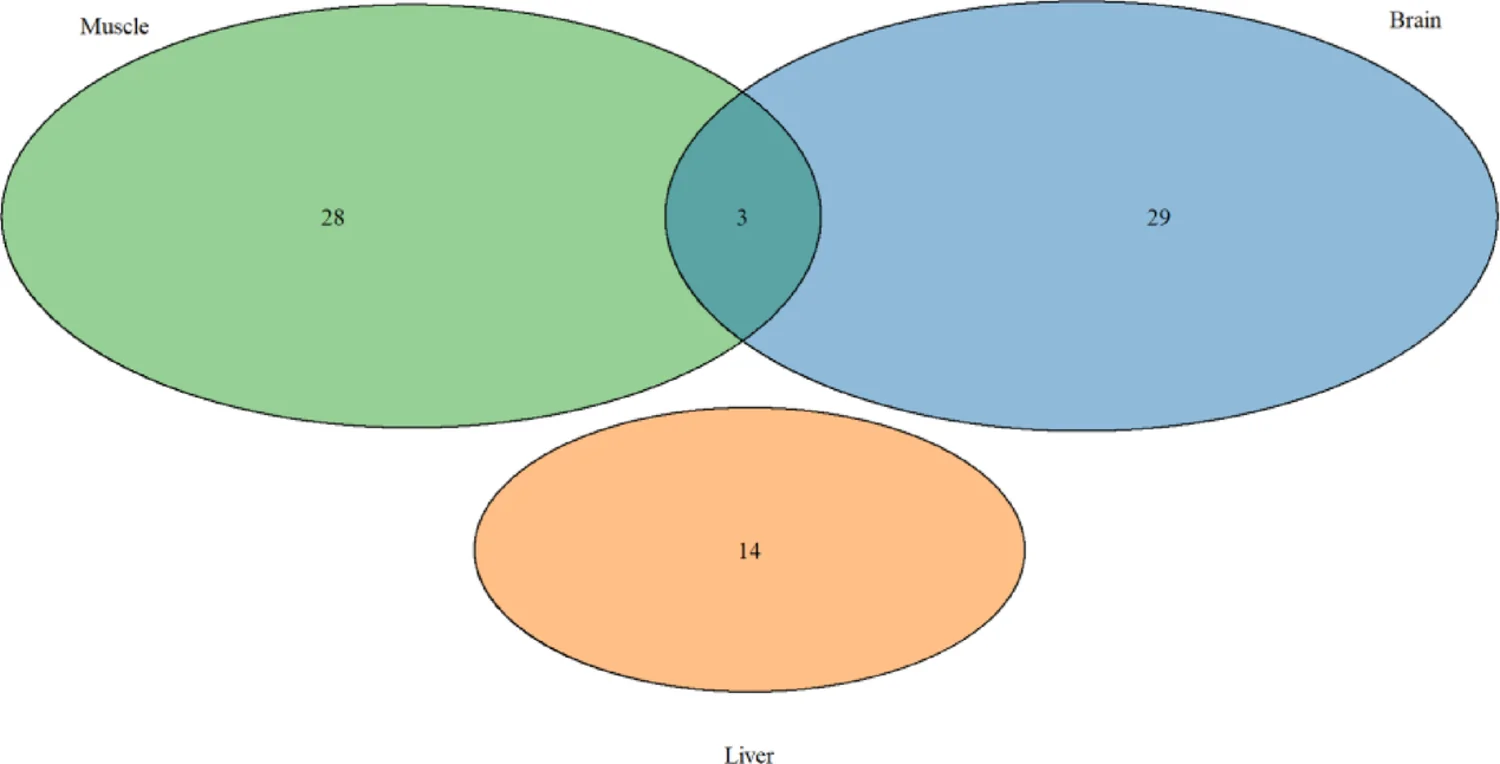
The Venn diagram presents the non-annotated and annotated candidate genes in each tissue

We found some AS events for the three tissues, as shown in Fig. 2 (Supplementary Table S2). For brain and skeletal muscle, more candidate genes were related to alternative first exon (AF), followed by skipped exon (SE). Beyond these events, it is possible to observe that more candidate genes were annotated for AF in the brain and more non-annotated candidate genes were found for AF in the skeletal muscle. In the SE event, the brain showed approximately the same number of annotated and non-annotated candidate genes, whereas a large number of non-annotated candidate genes were identified in the skeletal muscle. In liver tissue, both SE and RI events presented more candidate genes, followed by the AF event. The other events could be considered rare for this comparison between SOY1.5 and SOY3.0, which presented fewer AS local events with a significant FDR. The Venn diagram showed all the candidate genes for the three tissues (Fig. 3). We could also observe an interaction among three candidate genes between brain and skeletal muscle tissues, whereas liver showed no interaction (Fig. 3).

**Fig. 4:**
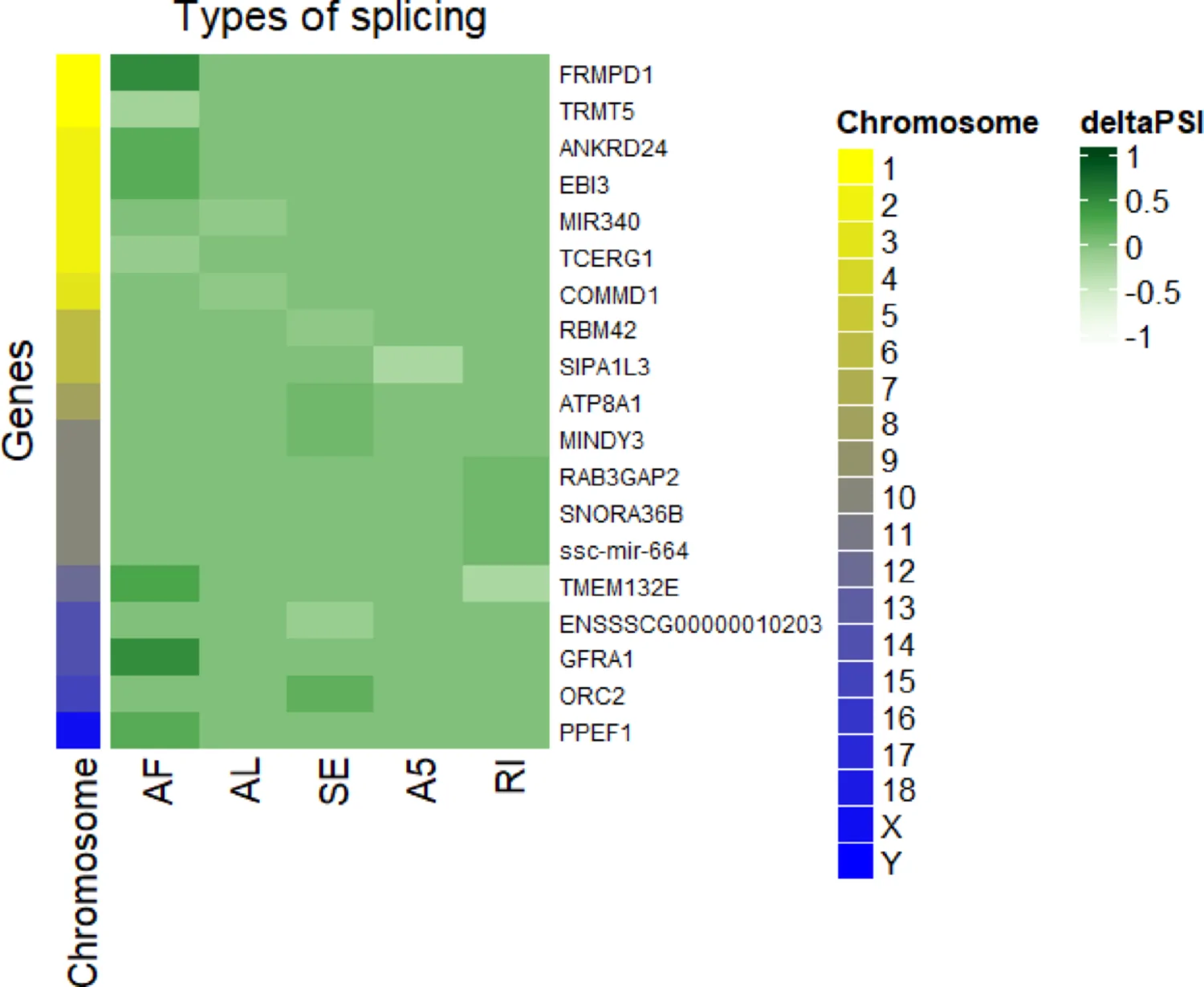
The heatmap illustrates differential splicing candidate genes in the brain, where events such as *AF* alternative first exon, *AL* alternative last exon, *SE* skipped exon, *A5* alternative 5’ splice-site, and *RI* retained intron are highlighted. The dark green means a delta PSI close to 1 and light green means a delta PSI close to -1. The chromosome column shows the location of each gene in the pig genome

The description of each candidate gene was based on the highest DPSI value, which, as previously explained, indicates the frequency of differential splicing in each diet. In the brain, each event can be viewed by the heatmap in Figure 4. For SE event, the genes found were *RBM42* (RNA Binding Motif Protein 42), *ATP8A1* (RNA Binding Motif Protein 42), *MINDY3* (MINDY Lysine 48 Deubiquitinase 3), *ORC2* (Origin Recognition Complex Subunit 2), and *ENSSSCG00000010203* (Heterogeneous Nuclear Ribonucleoprotein F). All of them were protein-coding, and their annotation is described in Supplementary Table S1. *ORC2* presented the highest positive DPSI of 0.18, and this gene is associated with the DNA replication process. This SE event in *ORC2* is favorable to occur in SOY1.5. However, this event is not occurring in the protein-coding region, given that the AS is located in exon 3, and this exon is part of the 5’ UTR.

For the RI event, the *RAB3GAP2* and *TMEM132E* candidate genes are protein-coding. The *TMEM132E* (Transmembrane Protein 132E) gene showed a DPSI of -0.27 and is present in pigs fed SOY3.0; it encodes a membrane protein. All the annotated candidate genes with the RI event can be viewed in Supplementary TableS1. Two transcript variants for the *TMEM132E* gene were found in Ensembl. Exon 10 presents an RI of 28 bp. Nonetheless, no differences were found in the protein sequence of these two variants using BLASTp, given that both isoforms have the same size in the tenth exon (Supplementary Figure S1).

The mutually exclusive exons (MX) event had just one non-annotated gene (FDR ≤ 0.1) (Supplementary Table S2), whereas the alternative last exon (AL) presented four genes, two non-annotated and two annotated (Fig. 2). One annotated gene was protein-coding, and the other was a miRNA. The RNA splicing is different for miRNA; thus, we decided to present the protein-coding *COMMD1* (Copper Metabolism Domain Containing 1). For this gene, its DPSI was equal to -0.08, indicating an occurrence in pigs fed SOY3.0. The *COMMD1* gene has a total of three exons in the two annotated variants. Transcript 202 of this *COMMD1* gene possesses this alternative splicing. The AL event shows an alternative that allows the CDS region to end in exon 3. When the AL occurs, the CDS region ends in an intronic region, containing a total of 20 bp in intron 2–3. The protein sequence showed some differences, but none are located in the splicing region (Supplementary Figure S2).

The alternative first exon (AF) event occurs specifically in the non-CDS region. Thus, this AF event might influence gene expression. Moreover, the *FRMPD1* protein (FERM and PDZ Domain Containing) is involved in regulating the G protein-coupled receptor signaling pathway and encodes two variants. No 3D protein structure was detected in the UniProt database. Transcript 202 presented a 31-aa deletion and a 4-aa replacement in both transcripts according to the Blastp tool (Supplementary Figure S3).

The alternative 5’ splice-site (A5) event occurs in the same region on both transcripts (Supplementary Table S1). One part of the intron 15–16 (35 bp) is retained on the alternative 2, whereas the alternative 1 totally cuts the intron 15–16. In the protein sequence of the *SIPA1L3* gene (signal-induced proliferation associated 1 like 3) presented a glutamine (Q) in exon 19 (1497 aa) of transcript 201, whereas the transcript 202, of this same gene, had a deletion of 1179 aa between exons 16 and 17 (Supplementary Figure S4).

**Fig. 5:**
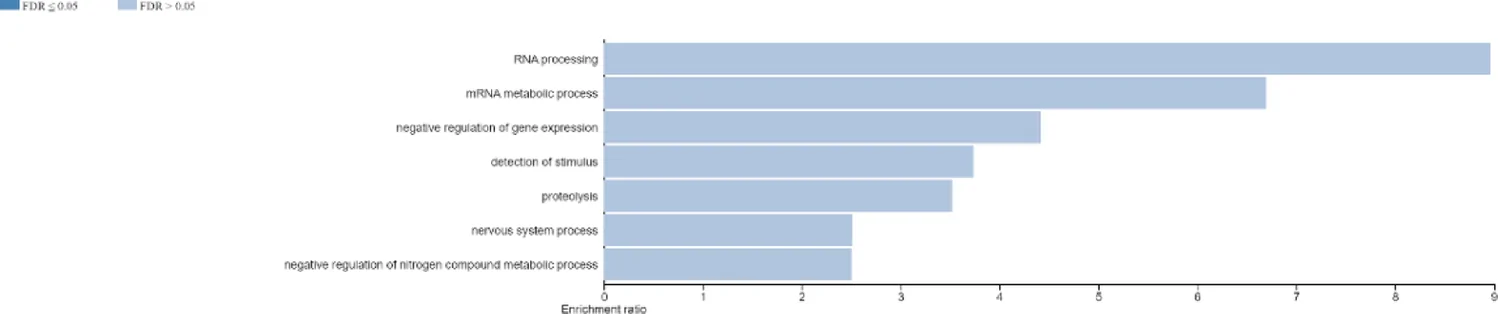
Functional enrichment was carried out by WebGestalt and biological processes GO terms can be viewed for 20 annotated genes in the pig brain

The 20 annotated genes were enriched for biological process GO terms, as shown in Fig. 5. All terms had an FDR > 5%, but the most significantly enriched term was “RNA processing”. Taken together, these results highlight that AS occurs not only in the CDS region but also in the 5’UTR in the brain. This finding may relate to the brain’s potential role in controlling metabolic pathways or regulating processes in the whole organism, such as in the liver and skeletal muscle.

**Fig. 6:**
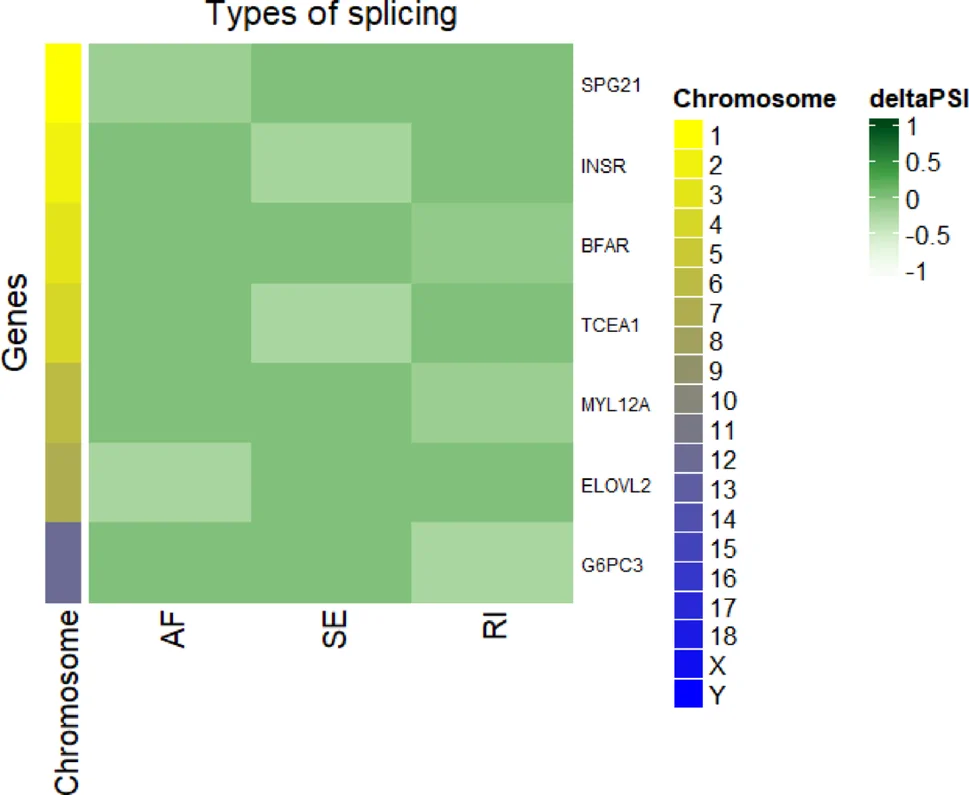
The heatmap illustrates differentially spliced genes in the liver; events such as *AF* alternative first exon, *SE* skipped exon, and *RI* retained intron are highlighted in each gene. Dark green means a delta PSI close to 1, and light green means a delta PSI close to -1. The chromosome column shows each gene’s location in the pig genome

To better understand AS events in the liver, we observed local events; we identified 21 candidate genes in liver tissue: 14 non-annotated and 7 annotated by Ensembl v.115 (*Sus scrofa* 11.1), as shown in Supplementary Table S2 and Fig. 2. The first event was the SE event, which included two genes annotated by Ensembl (v.115 – *Sus scrofa* 11.1): *TCEA1* (Transcription Elongation Factor A1) and *INSR* (Insulin Receptor). We can observe genes presenting AS in pigs fed SOY3.0, given that only negative delta PSI values were identified in the heatmap (Fig. 6). All of these candidate genes are protein-coding, and their annotated regions are listed in Supplementary Table S1. *TCEA1*, which showed the highest DPSI, is associated with positive regulation of transcription mediated by RNA polymerase II. This candidate gene presented an SE event and is likely to occur in SOY3.0. This candidate gene also had five transcripts, and transcript 204 is the only isoform that showed a skipped exon 2 and a CDS region, as shown in Supplementary Figure S5.

The RI event had three genes annotated by Ensembl (Supplementary Table S2 and Fig. 2), and the gene *G6PC3* (Glucose-6-Phosphatase Catalytic Subunit 3) had a higher DPSI than the other genes for this event (DPSI = -0.25 – Fig. 6). There is one transcript for this gene, and the retained intron is 168 bp of intron 1–2. However, this region is not a CDS because this protein-coding region starts in the middle of exon 2. Therefore, this splicing suggests modulation of gene expression without modifying the structure of the protein.

Another splicing event was observed in the *ELOVL2* gene (ELOVL Fatty Acid Elongase 2), which is involved in fatty acid elongation and polyunsaturated and very long-chain fatty acid biosynthesis, and it has five transcripts. 

The AF event occurs specifically in the non-CDS region of transcripts 201 and 202, so it could also influence the gene expression of these transcripts (Supplementary Figure S6A). The splicing in transcript 203 is occurring either in the 5’ upstream sequence or exon 1. The CDS region is only 3 bp of the 94 bp in exon 1; thus, this AS also occurs in the non-CDS region (Supplementary Figure S6B). Transcripts 204 and 205 are isoforms, which have one alternative starting from exon 2 up to exon 4, and another alternative starting from intron 2–3 and going to exon 4 (Supplementary Figure S6C).

**Fig. 7:**
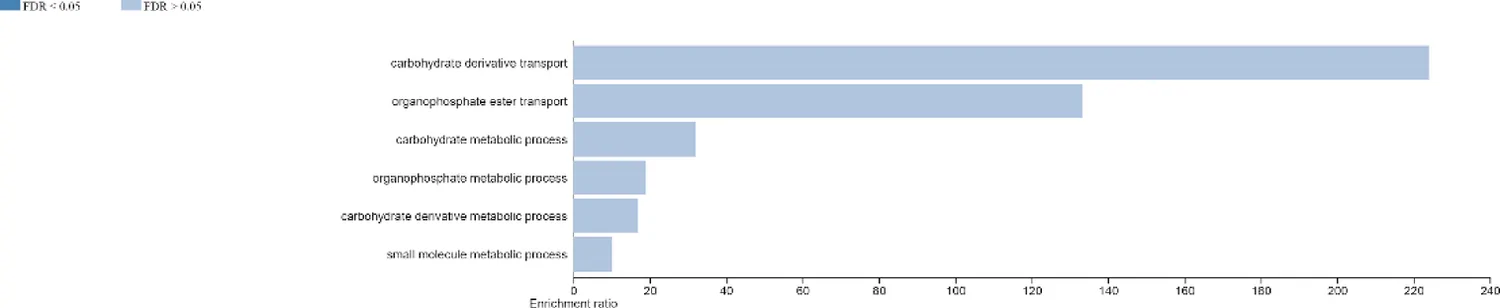
Functional enrichment was carried out by WebGestalt, and biological process GO terms can be viewed for seven annotated genes in the pig liver

Overall, the liver tissue presented less AS compared to the brain. This could be related to its essential role in controlling the metabolism of what will be either absorbed or eliminated by the organism. In addition, transcription could also be modified in all the processes of the liver. Important genes were associated with splicing: one for glucose metabolism and another for fatty acid elongation, suggesting that varying soybean oil concentration in the diet could influence lipid metabolism. The functional enrichment presented six biological process GO terms, and the most significant was “carbohydrate derivative transport”, as seen in Fig. 7.

**Fig. 8:**
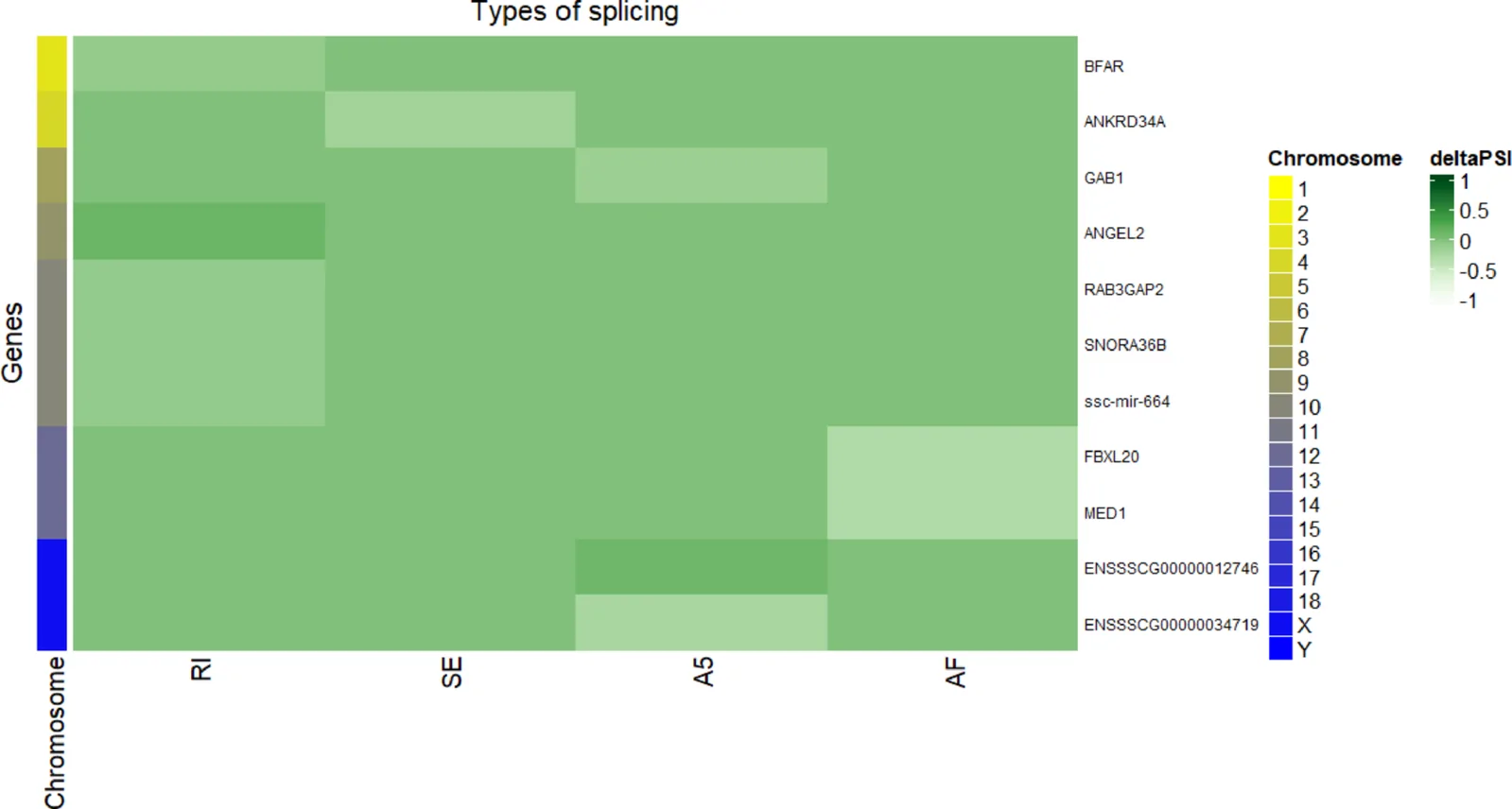
The heatmap illustrates differentially spliced genes in skeletal muscle; events, such as *AF* alternative first exon, *SE* skipped exon, *A5* alternative 5’ splice site, and *RI* retained intron, are highlighted in each gene. Dark green means a delta PSI close to 1, and light green means a delta PSI close to -1. The chromosome column shows the location of each gene in the pig genome

In skeletal muscle, lipid metabolism may also play an important role. Skeletal muscle is responsible for pigs’ growth performance during the growing-finishing phase. The AS events consisted of 37 genes in the skeletal muscle tissue (*L. lumborum*), of which 26 were non-annotated, and 11 were annotated by Ensembl *Sus scrofa* 11.1, as shown in Fig. 2 (Supplementary Table S2). The heatmap illustrates AS events in skeletal muscle, showing that AS generally occurs in pigs fed SOY3.0, with nine candidate genes with negative DSPI (Fig. 8). The SE event included one annotated candidate gene from Ensembl (v.115 – *Sus scrofa* 11.1). It was the *ANKRD34A* gene (Ankyrin Repeat Domain 34 A). This candidate gene is protein-coding, and its annotation is shown in Supplementary Table S1. The *ANKRD34A* gene has three transcripts. The SE event occurs in transcript 203. The CDS region starts in the last exon of this transcript. However, the SE event is not compromising the protein sequence for this transcript.

Presenting the RI event, which consisted of five annotated candidate genes by Ensembl (v.115 – *Sus scrofa* 11.1) (Fig. 2). These candidate genes were *RAB3GAP2* (RAB3 GTPase Activating Non-Catalytic Protein Subunit 2), *ANGEL2* (Angel Homolog 2), *SNORA36B* (*snoRNA)*,* ssc-mir-664* (miRNA), and *BFAR* (Bifunctional Apoptosis Regulator). The *RAB3GAP2*,* ANGEL2*, and *BFAR* candidate genes were protein-coding, and their annotations are listed in Supplementary Table S1. The *ANGEL2* gene presented four transcripts. The retained intron occurs inside exon 2 (94 bp) for transcripts 202 and 204. The CDS region starts in exon 2 for both transcripts. Thus, RI could compromise the protein sequence for transcripts 202 and 204 (Supplementary Figure S7). The transcripts 201 and 203 presented a CDS region at the end of exon 2 and in exon 3, respectively.

In contrast, the AF event consisted of two annotated genes by Ensembl (v.115 – *Sus scrofa* 11.1) (Fig. 2). They were the *MED1* (Mediator Complex Subunit 1) and *FBXL20* (F-Box and leucine rich repeat protein 20) genes with AS. All of them are protein-coding and were annotated in Supplementary Table S1. The *MED1* gene presented one transcript. There are two alternative splicing events; the former starts at -1 bp of exon 1, or could happen in intron 1–2. The latter starts at + 10 bp of exon 1, or could happen in intron 1–2. The CDS region starts at 200 bp of exon 1. Therefore, either − 1 bp or + 10 bp of exon 1 is not CDS, so these splicing regions could not interfere with the protein structure.

Based on the A5 event, which consisted of three annotated candidate genes by Ensembl (v.115 – *Sus scrofa* 11.1) (Fig. 8). They were the *GAB1* (GRB2 Associated Binding Protein 1), *ENSSSCG00000012746*, and *ENSSSCG00000034719* genes. All of them are protein-coding and were annotated in Supplementary Table S1. The *GAB1* gene presented two transcripts. One transcript includes exon 5 and intron 4–5 (represented by 2 bp), while the other includes exon 5 and exon 4. In exon 5, there is an insertion of valine in transcript 201, and this residue is deleted in transcript 202 (Supplementary Figure S8).

**Fig. 9:**
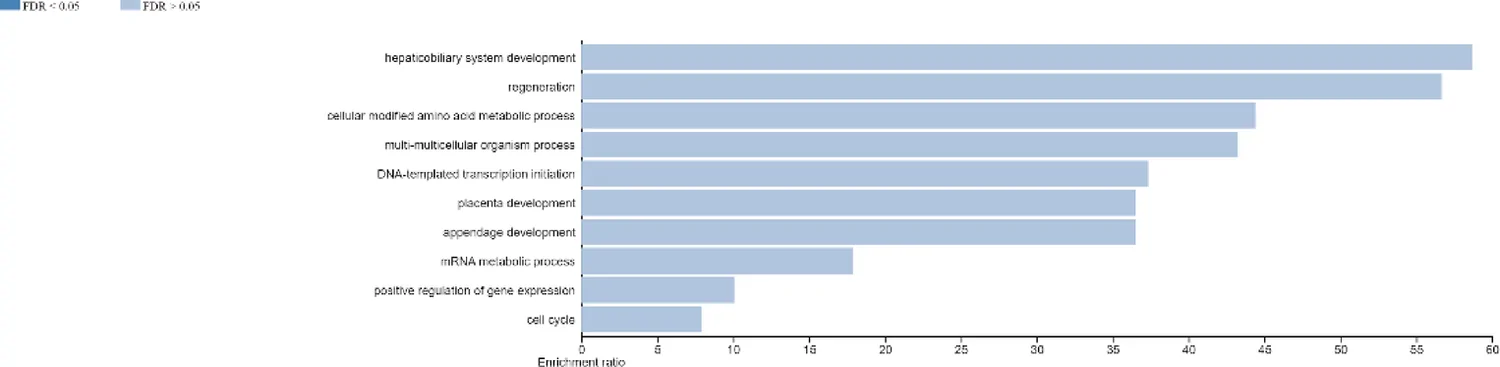
Functional enrichment was carried out by WebGestalt, and biological process GO terms can be viewed for eleven annotated candidate genes in the pig skeletal muscle

The candidate genes presented in skeletal muscle were actually related to the plasma membrane and transcription-related RNA polymerase. These functions in the cell are related to receiving signals and regulating gene expression inside the muscle. The functional enrichment presented ten biological process GO terms, and the most significant was “hepaticobiliary system development,” followed by the “regeneration” biological process GO term, as seen in Fig. 9. Thus, exploring candidate genes with AS among these three different tissues and observing the modification of protein structure or candidate gene regulation through local splicing regions in genes suggest that the AS could be an important mechanism for these three tissues of pigs.

## Discussion

First, we planned to discuss the relative importance of most annotated genes for certain tissues compared to others. In addition, we focused on the 5’ UTR as a key element in AS. Another topic was addressed based on the RNA strand (negative or positive). A discussion was also held regarding lipid metabolism and how the regulation of RNA splicing can modulate this metabolism. Furthermore, the importance of all the tissues studied was considered, revealing their role in AS.

In the three tissues, we have AS occurring in the 5’ UTR, i.e., *ORC2* and *FRMPD1* in the brain, or *G6PC3* and *ELOVL2* in the liver, or *ANKRD34A* and *MED1* in the skeletal muscle. However, we also found AS in the CDS region of all the tissues, such as *TMEM132E* for the brain, *TCEA1* for the liver, or *ANGEL2* for skeletal muscle. We also have introns retained in the brain, such as *COMMD1* and *SIPA1L3*, or *GAB1* in the skeletal muscle. All of these events can suggest how AS is supporting pigs fed SOY1.5 or SOY3.0.

The brain and muscle tissues present specific structures and conserved patterns of AS because both tissues possess highly conserved repertoires of AS events observed in vertebrates (Raj and Blencowe 2015). Pigs fed SOY1.5 presented splicing on *ORC2* and *FMRPD1* genes in the brain. The AS in the brain could be attractive for regulating gene activity through the production of nonfunctional protein isoforms (Daoud et al. 1999a; Keifer 2021; Liu et al. 2021). In the liver, the AS contributes to the pathogenesis of liver diseases; its role appears less central in the liver than in other organs. The genes in this study were only related to SOY3.0 (only negative DPSI values). Taking the fibronectin EDA exon as an example, while certain isoforms may promote liver fibrosis, the modulation of this exon has a much more significant impact on atherosclerosis and pulmonary fibrosis, suggesting a comparatively limited direct impact on hepatic tissue (Baralle and Baralle 2021). The *ANGEL2* gene had a strong splicing response in skeletal muscle in pigs fed SOY1.5. Skeletal muscle has been the first tissue in which AS is widely observed, helping to regulate proteins involved in ion transport, structural functions, and excitation-contraction coupling (Daoud et al. 1999b; Llorian and Smith 2011; Cai et al. 2024). The regulatory mechanisms of AS are well understood, but the consequences of specific spliced isoforms and their networks remain uncertain (Baralle and Giudice 2017). In general, all the tissues analyzed play an important role in neuroplasticity, metabolism, and muscle protein growth in pigs.

The coding exons of genes constitute 10% of the human genome and contain introns and UTRs, which are sites where AS can occur (Kim et al. 2018). An important region that can control gene expression and translation patterns is the 5’ UTR. For the liver, the SOY3.0 diet presented high negative DPSI values, showing that these involved genes were regulated by the 5’ UTR, except for the *TCEA1* gene, which had SE in exon 2, modifying the CDS region. The 5’ UTR can also be responsible for pathological diseases, since AS is present and dysregulated, such as colorectal cancer, Alzheimer’s disease, etc. (Ryczek et al. 2023). The other two tissues also presented genes regulated by 5’ UTR; moreover, intron retention was modifying these CDS regions in both tissues. The intronic splice enhancer (ISE) in the 5’ UTR can affect gene expression in plant and animal organisms, especially in specific genes (Ohta et al. 2018). The presence of upstream ORFs (uORFs) in the 5’ UTR can regulate AS and consequently alter the levels of translation of some genes that present this pattern, since in this case the ribosome scans the first AUG in the uORF region, and this scanning impairs the translation of the entire mRNA (de Breyne et al. 2025).

Antisense transcription is a crucial feature in the human genome, where antisense RNA transcripts tend to undergo fewer splicing events and exhibit lower abundance levels than sense transcripts (Wright et al. 2023). Pigs fed SOY3.0 generally showed more splicing events in the negative strand than pigs fed SOY1.5 (24 genes from a total of 38 genes). There is a strong functional correlation between antisense transcripts and AS complexity in sense-antisense pairs, with studies reporting enrichment of alternative exons and an increased number of isoforms in overlapping regions (Kung et al. 2013; Celik et al. 2025). For instance, the *HTTAS* transcript, located on the antisense strand, illustrates this capacity, as it presents spliced variants that regulate the levels of the sense transcript (Mills et al. 2016). The brain tissue had more AS in SOY1.5 (12 genes) compared to SOY3.0 (eight genes), whereas the skeletal muscle presented just two genes in the SOY1.5 diet and nine genes in the SOY3.0 diet, and the liver did not present any AS in pigs fed SOY1.5, but seven genes in the SOY3.0 diet. As far as we know, antisense RNA can bind to the sense pre-mRNA, masking local spliceosome sites and modifying the balance between processing variants (Faghihi and Wahlestedt 2009; Dominic Mills et al. 2013; Jeon et al. 2024). Even though antisense RNAs may contain fewer introns than protein-coding mRNAs (Kung et al. 2013; Jeon et al. 2024), their interaction with the sense strand is critical for transcriptome diversity through AS, especially in complex tissues such as the brain (Mills et al. 2016).

A fat diet is not only associated with energy gain but is also associated with lipid homeostasis (Li et al. 2021). Alterations in the linoleic and alpha-linolenic acid ratio can affect lipid metabolism gene expression, especially increasing the linoleic and alpha-linolenic acid ratio, leading to progressive obesity (Wang and Wang 2023). In this context, the SOY3.0 diet presented a higher n-6/n-3 ratio compared to SOY1.5. Fatty acids can be regulated by several isoforms from two genes in mice: *ACSL* and *FATP;* despite their different locations in cells, these isoforms can channel fatty acids into several metabolic pathways (Bu et al. 2009; Bovo et al. 2025). In the obese human model, some genes regulating RNA processing also show decreased expression in the liver and skeletal muscle, including the *SFRS10* gene, which affects *LPIN1* splicing and may increase lipogenesis (Pihlajamäki et al. 2011; Liu et al. 2024).

Although the SOY3.0 diet showed more AS events than SOY1.5, our results do not allow us to conclude that the higher soybean oil level directly caused the increase in isoform abundance. Nevertheless, dietary fatty acids suggest modulate metabolic pathways sensitive to cellular lipid status, potentially influencing AS dynamics. Thus, candidate genes involved in lipogenesis or fatty acid metabolism might present specific variants in response to the higher lipid intake observed in the SOY3.0 diet.

Finally, considering that frontal lobe of brain can control several processes related to liver and skeletal muscle, on the other hand, liver is specifically metabolizing all the substances produced by muscular growth and nervous molecules, whereas the skeletal muscle is in development and growth, the nervous system needs to adapt to feed these muscles, whereas the liver works for nutrition and excretion of (un)desirable substances.

## Conclusion

Our findings searched for the hypothesis that varying levels of soybean oil in the diet might modify the AS profile in pigs. However, further studies, including functional validation of isoforms, protein quantification, and cellular-level analyses, are needed to confirm the causal relationship between lipid intake and splice modulation. Thus, understanding how nutrients do not only modulate RNA processing in metabolically distinct tissues but also expanding the use of the pig as a biomedical model and contributing to the development of innovative therapeutic strategies targeting diseases in which AS plays a central role.

## Supplementary Information

Below is the link to the electronic supplementary material.

Supplementary Material 1

Supplementary Material 2

Supplementary Material 3

Supplementary Material 4

Supplementary Material 5

Supplementary Material 6

Supplementary Material 7

Supplementary Material 8

Supplementary Material 9

Supplementary Material 10

Supplementary Material 11

## Data Availability

The dataset used in this study is available in the European Nucleotide Archive (ENA) repository (EMBL-EBI) under accession PRJEB50513, PRJEB52629, and PRJEB52665: [https://www.ebi.ac.uk/ena/browser/view/PRJEB50513], [https://www.ebi.ac.uk/ena/browser/view/PRJEB52629], and [https://www.ebi.ac.uk/ena/browser/view/PRJEB52665].

## Code availability

The raw dataset used in this study is available in the European Nucleotide Archive (ENA) repository (EMBL-EBI) under accession PRJEB50513, PRJEB52629, and PRJEB52665: [https://www.ebi.ac.uk/ena/browser/view/PRJEB50513], [https://www.ebi.ac.uk/ena/browser/view/PRJEB52629], and [https://www.ebi.ac.uk/ena/browser/view/PRJEB52665].
