# Supplementary material for: Alternative splicing dynamics in pig tissues under soybean oil supplementation
Source: Mamm Genome. 2026 Oct 15;37(1):111. doi: 10.1007/s00335-026-10279-4 (PMC13672616; doi:10.1007/s00335-026-10279-4)
Supplement: Supplementary file 1 [file 335_2026_10279_MOESM11_ESM.docx]

**Supplementary Figure S1** Transcript 201 is colored in blue, and transcript 202 is colored in red. The exon 10 is presented in yellow and white colors. The retained intron occurs after 421 bp in the exon 10. Sources: UniProt and ChimeraX

**Supplementary Figure S2** Region in blue indicates the 31-aa deletion and red region identifies the 4-aa replacement in Blastp tool

**Supplementary Table S3** Transcript 1 is colored in blue and presented insertion of three residues. Transcript 2 is colored in red and presented four replaced residues. Source: UniProt and ChimeraX

**Supplementary Figure S4** The protein blue is represented by transcript 201, and the protein red is represented by transcript 202. The glutamine (GLN - Q) can be viewed in transcript 201 on residue 328, and a serine (SER – S) is located on residue 466 in transcript 202 in the red color. Source: UniProt and ChimeraX

**Supplementary Figure S5** The colored region corresponds to exon 2, which could be skipped in pigs fed 3.0% soybean oil. This is the 3D protein structure of transcript 204

**Supplementary Figure S6** (A) The protein colored in blue is transcript 201, and the protein colored in red is transcript 202. The cyan region is an insertion of several residues that were not present in transcript 201. The yellow lines represent the exact correspondence between the residues in transcripts 201 and 202. Sources: UniProt and ChimeraX. (B) Two models of proteins for the same transcript 203, colored in blue and red. The yellow region represents exons 1 and 3. The AS starts in the end of exon 1 and goes to exon 3 instead of the 5’ upstream sequence. (C) Four models of proteins, the blue and magenta models represent transcript 204, and the red and yellow models represent transcript 205. Regions colored in white represent exon 2, and cyan regions represent exon 4

**Supplementary Figure S7** The protein models blue (transcript 202) and magenta (transcript 204) represent the non-retained intron at exon 2. Whereas the protein models red (transcript 202) and yellow (transcript 204) represent the exact intronic region, colored in white and brown, respectively. Sources: UniProt and ChimeraX

**Supplementary Figure S8** Protein model related to transcript 201 is colored in blue, and presents a residue of valine in position 251, whereas protein model related to transcript 202 is colored in red, and presents a residue of serine in position 251. The region colored in magenta represents the same region colored in yellow and cyan. Sources: UniProt and ChimeraX
